# Cerebral venous sinus thrombosis in pregnancy and the postpartum period: epidemiology, management, and maternal mortality—a systematic review and meta-analysis

**DOI:** 10.3389/fneur.2026.1836450

**Published:** 2026-09-11

**Authors:** Tayseer Al Muteri, Rimah Dhayfallah Almutairi

**Affiliations:** 1 Department of Obstetrics and Gynaecology, College of Medicine, Majmaah University, Majmaah, Saudi Arabia; 2 College of Medicine, Qassim University, Buraydah, Saudi Arabia

**Keywords:** anticoagulation, cerebral venous sinus thrombosis, cerebral venous thrombosis, maternal mortality, meta-analysis, postpartum, pregnancy, puerperium

## Abstract

**Background:**

Cerebral venous sinus thrombosis is a rare cerebrovascular disease of importance both during pregnancy and in the puerperium. However, the existing cohort literature relates to two distinct clinical populations: women with acute onset of pregnancy−/puerperium-associated cerebral venous thrombosis (CVST), and women with known previous CVST who were assessed in connection with a subsequent pregnancy. As these populations address distinct clinical questions and have distinct denominators and follow-up structures, this review has distinguished outcomes from the two populations and synthesized them separately.

**Methods:**

A PRISMA-compliant systematic review was conducted searching six bibliographic databases for evidence on CVST/central venous thrombosis (CVT), pregnancy/puerperium, and follow-up cohorts. Cohort studies only have been eligible for inclusion, with the additional criterion of a follow-up period of ≥5 years or participant-level follow-up period of ≥5 years. This particular criterion was used mainly as an aid to interpreting data on recurrence or subsequent pregnancy, while for outcomes related to acute obstetric CVST, it was used simply as a measure of study period. Outcomes have been summarized as pooled proportions by random-effects meta-analysis with pre-specified stratification in follow-up cohorts (women with known CVST in the past) and study period cohorts (acute obstetric CVST).

**Results:**

Twelve studies were included. In follow-up cohorts assessing subsequent pregnancy following previous CVST, deliveries/livebirths pooled to 69.6% (95% CI 59.2 to 78.4%, I^2^ = 51.7%), spontaneous miscarriage pooled to 19.4% (95% CI 13.8 to 26.5%, I^2^ = 20.6%), and recurrence of cerebral venous sinus thrombosis during subsequent pregnancy/puerperium pooled to 3.17% (95% CI 1.43 to 6.89%, I^2^ = 0%). In acute pregnancy/puerperium cerebral venous sinus thrombosis cohorts, maternal mortality pooled to 8.28% (95% CI 5.65 to 11.97%, I^2^ = 0%), while acute-phase anticoagulation use pooled to 92.9% (95% CI 64.8 to 98.9%) with high heterogeneity (I^2^ = 81.2%). These outcomes were thus interpreted within the context of their particular population and observation period.

**Conclusion:**

Long-term follow-up cohorts indicated that subsequent pregnancy following previous cerebral venous sinus thrombosis was associated with good delivery outcomes and low recurrence in cases when CVST management included structured risk stratification and appropriate prophylaxis. Acute obstetric CVST cohorts, on the other hand, dealt with acute morbidity, mortality, and treatment, and not with future pregnancy safety, with significant maternal mortality burden.

**Systematic review registration:**

PROSPERO registration number CRD420261328921.

## Introduction

Cerebral venous sinus thrombosis (CVST) represents a relatively unusual but clinically significant cause of stroke due to thrombotic occlusion of the dural venous sinuses and/or cerebral veins. In this manuscript, CVST was used as the preferred umbrella term for obstetric cerebral venous sinus thrombosis; the abbreviation CVT was retained only where the original studies or cited literature used the broader term cerebral venous thrombosis. This occlusion initiates a pathophysiologic cascade marked by impaired venous drainage, raised intracranial pressure, venous congestion, and, in severe cases, hemorrhagic venous infarction or intracranial hemorrhage. Although the overall incidence of CVST in the general population is low, pregnancy and the puerperium create a transient prothrombotic risk environment in which physiologic hemostatic changes may predispose susceptible women to cerebral venous occlusion (1–3).

Pregnancy and the puerperium are characterized by dynamic changes in coagulation, fibrinolysis, blood volume, and vascular tone, which together create a physiologic hypercoagulable state. This physiologic adaptation likely evolved to minimize hemorrhage at delivery but simultaneously increases the risk of venous thromboembolism in susceptible individuals. Other pregnancy-specific exposures, including hypertensive disorders of pregnancy, cesarean delivery, infection, dehydration, anemia, stillbirth, and immobility, may contribute synergistically to thrombotic risk through endothelial injury, inflammation, hemoconcentration, placental vascular pathology, and peripartum venous stasis (1–5).

Contemporary guidelines from the American Heart Association/American Stroke Association and the European Stroke Organization endorse anticoagulation as the cornerstone of CVST treatment, typically using heparin-based regimens and continuing treatment even in selected patients with intracranial hemorrhage when the clinical context supports prevention of thrombus propagation and recanalization (1, 2). Nevertheless, practice variation persists across individual cohorts and regions with respect to anticoagulation timing, intensity, duration, use of advanced interventions in severe cases, and approaches to thrombophilia testing and secondary prophylaxis (6–10). These uncertainties remain particularly relevant when counseling women after an index CVST about the safety of subsequent pregnancy, expected obstetric outcomes, and the balance of benefits and risks associated with prophylactic anticoagulation during gestation and the postpartum period.

Despite an expanding literature base, evidence synthesis in obstetric CVST remains challenged by clinical and methodological heterogeneity. A central issue is that two clinically different populations are often discussed together: women who present with acute pregnancy−/puerperium-associated CVST, for whom the immediate questions involve diagnosis, anticoagulation, functional outcome, and maternal survival; and women with a previous history of CVST who subsequently become pregnant, for whom the relevant questions concern recurrence, miscarriage, live birth, and prophylaxis during a future pregnancy. These populations differ in outcome timing, denominator structure, and follow-up requirements; therefore, pooled estimates require explicit separation by analytic population and assessment window.

Therefore, this systematic review and meta-analysis was undertaken to synthesize cohort evidence about CVST in pregnancy and the postpartum period using an explicitly separated two-stratum framework: (i) follow-up cohorts of women with previous CVST evaluated for outcomes of subsequent pregnancy, and (ii) acute pregnancy−/puerperium-associated CVST cohorts evaluated for acute management, maternal mortality, and early functional outcomes. Quantitative pooling was restricted to endpoints with compatible population and time-base structures, and qualitative synthesis was used to contextualize timing, diagnostic confirmation, clinicoradiologic profile, prothrombotic architecture, and management heterogeneity. From a public health perspective, this distinction is essential because counseling about future pregnancy safety is not the same clinical question as estimating acute maternal mortality after obstetric CVST.

## Materials and methods

### Protocol and reporting

This systematic review and meta-analysis was planned *a priori* and conducted in accordance with the PRISMA 2020 reporting guidelines (11). The protocol prespecified eligibility criteria, outcomes, subgroup/sensitivity analyses, and statistical methods and was registered in PROSPERO (CRD420261328921). The PROSPERO record was reviewed against the completed manuscript, and the corresponding author will update the registry entry to reflect completion of the review, final search status, and the explicit separation of acute obstetric CVST cohorts from subsequent-pregnancy-after-prior-CVST cohorts. The review question was prespecified via a PECOS framework to ensure transparent eligibility criteria and reproducible study selection as mentioned below:

- The Population consisted of women of reproductive age who either (i) developed cerebral venous sinus thrombosis (CVST) or cerebral venous thrombosis (CVT) during pregnancy or the postpartum/puerperal period or (ii) had a documented history of CVST/CVT and were subsequently assessed for pregnancy-related outcomes.
- The Exposure included pregnancy- and postpartum-associated physiological and obstetric context (including gestational and puerperal timing) and/or a prior CVST/CVT index event in cohorts followed through subsequent pregnancies.
- The Comparator was not required for single-arm event-rate synthesis; where internal comparisons were available, comparators involved pregnancy versus postpartum strata, prophylaxis versus no prophylaxis, and thrombophilia/hypertensive disorder strata.
- The Outcomes were prespecified to correspond to the review aim: incidence proxies/event rates within cohorts, management patterns (anticoagulation and related care processes), maternal mortality and functional outcomes, thrombotic recurrence (recurrent CVST and extra-cerebral venous thromboembolism, defined as objectively diagnosed venous thromboembolic events outside the cerebral venous system, such as deep-vein thrombosis or pulmonary embolism), and key obstetric outcomes (live birth, miscarriage, stillbirth, preterm delivery, and placenta-mediated complications).
- The Study design was restricted to cohort studies (prospective or retrospective, single- or multicenter, or register-based).

### Inclusion/exclusion criteria

Inclusion criteria specified cohort studies enrolling either women with acute pregnancy- or puerperium-associated CVST/CVT or women with a previous CVST/CVT who were assessed for subsequent pregnancy outcomes, with objective clinical/imaging confirmation of CVST/CVT as defined by the study. Studies were eligible when they had either a cohort-level assessment period of ≥5 years or participant-level longitudinal follow-up of ≥5 years. The ≥5-year threshold was chosen primarily to capture recurrence and reproductive outcomes that accrue over years and to distinguish this review from prior syntheses dominated by short-duration cohorts. For acute outcomes such as maternal mortality, in-hospital functional status, and anticoagulation use, this criterion was applied as a cohort study-period requirement, not as a biological assumption that acute endpoints require long follow-up. This design choice may have excluded informative short acute series and may preferentially select centers with stronger surveillance or registry infrastructure; therefore, it was considered during interpretation and certainty assessment.

Exclusion criteria encompassed non-cohort designs, such as cross-sectional studies, case series without cohort follow-up structure, and case reports; studies without a clearly definable pregnancy/postpartum CVST/CVT cohort or prior-CVST cohort with pregnancy assessment; insufficient duration to meet the ≥5-year criterion; non-human or non-clinical investigations; or irretrievability in full text for eligibility verification and extractable outcome data.

### Search strategy

The search strategy was carried out in six databases to maximize sensitivity while maintaining construct specificity for CVST/CVT and obstetric timing. Controlled vocabulary particular to each database (e.g., MeSH/Emtree headings) was used, supplemented by free-text synonyms, proximity operators when possible, and Boolean logic. Search strings were iteratively refined to balance recall and precision, formatted to individual platform field tags and thesaurus mappings, and executed without geographic restriction (Table 1). Filters were limited to human studies, and, as required, study-design keywords emphasized cohort evidence while avoiding overly restrictive methodological hedges, which could diminish sensitivity.

**Table 1:** Search strings utilised across the included studies.

Database	Search string (database-adapted; no two identical)
PubMed/MEDLINE	((“Sinus Thrombosis, Intracranial”[MeSH] OR “Cerebral Vein Thrombosis”[MeSH] OR “intracranial venous thrombosis”[tiab] OR “cerebral venous thrombosis”[tiab] OR “cerebral venous sinus thrombosis”[tiab] OR CVT[tiab] OR CVST[tiab] OR “dural sinus thrombosis”[tiab] OR “cerebral sinus thrombosis”[tiab]) AND (“Pregnancy”[MeSH] OR “Puerperium”[MeSH] OR postpartum[tiab] OR puerper*[tiab] OR peripartum[tiab] OR pregnancy[tiab] OR gestation*[tiab] OR gravid*[tiab]) AND (cohort[tiab] OR “follow-up studies”[MeSH] OR longitudinal[tiab] OR registry[tiab] OR recurrence[tiab] OR mortality[tiab] OR anticoagulat*[tiab] OR heparin[tiab] OR “low molecular weight heparin”[tiab] OR enoxaparin[tiab] OR obstetric*[tiab] OR “live birth”[tiab] OR miscarriage[tiab] OR stillbirth[tiab] OR “preterm”[tiab]))
Embase (Emtree)	(‘cerebral vein thrombosis’/exp. OR ‘dural sinus thrombosis’/exp. OR ‘intracranial venous thrombosis’/exp. OR (cerebral NEAR/3 (venous OR vein* OR sinus*) NEAR/3 thrombosis):ti,ab OR CVST:ti,ab OR CVT:ti,ab) AND (‘pregnancy’/exp. OR ‘postpartum period’/exp. OR ‘puerperium’/exp. OR (postpartum OR puerper* OR peripartum OR gestation* OR gravid*):ti,ab) AND (‘cohort analysis’/exp. OR ‘prospective study’/exp. OR ‘retrospective study’/exp. OR ‘follow up’/exp. OR registry:ti,ab OR longitudinal:ti,ab) AND (mortality:ti,ab OR recurrence:ti,ab OR ‘venous thromboembolism’/exp. OR anticoagulant agent/exp. OR heparin/exp. OR ‘low molecular weight heparin’/exp. OR obstetric outcome/exp. OR live birth/exp. OR miscarriage/exp. OR stillbirth/exp. OR prematurity/exp)
Scopus	TITLE-ABS-KEY((“cerebral venous sinus thrombosis” OR “cerebral venous thrombosis” OR “intracranial venous thrombosis” OR “dural sinus thrombosis” OR CVST OR CVT OR “cerebral sinus thrombosis”) AND (pregnan* OR puerper* OR postpartum OR peripartum OR gestation* OR gravid*) AND (cohort OR longitudinal OR “follow up” OR registry OR retrospective OR prospective) AND (mortality OR death OR recurrence OR “recurrent CVST” OR “venous thromboembolism” OR VTE OR anticoagulat* OR heparin OR enoxaparin OR obstetric* OR “live birth” OR miscarriage OR stillbirth OR preterm))
Web of Science Core Collection	TS = ((“cerebral venous” NEAR/3 (thrombosis OR thrombus*) OR “venous sinus” NEAR/3 thrombosis OR “dural sinus” NEAR/3 thrombosis OR CVST OR CVT) AND (pregnan* OR postpartum OR puerper* OR peripartum OR gestation* OR gravid*) AND (cohort OR follow-up OR longitudinal OR registry OR “population-based”) AND (mortality OR fatal* OR recurrence OR “recurrent” OR anticoagulat* OR heparin OR “low molecular weight heparin” OR obstetric* OR miscarriage OR stillbirth OR “live birth” OR preterm))
Cochrane CENTRAL	((“cerebral venous thrombosis” OR “cerebral venous sinus thrombosis” OR “dural sinus thrombosis” OR CVT OR CVST):ti,ab,kw) AND ((pregnan* OR postpartum OR puerper* OR peripartum OR gestation*):ti,ab,kw) AND ((cohort OR follow-up OR longitudinal OR registry OR observational):ti,ab,kw) AND ((anticoagul* OR heparin OR enoxaparin OR mortality OR recurrence OR “venous thromboembolism” OR obstetric OR miscarriage OR stillbirth OR “live birth”):ti,ab,kw)
CINAHL (EBSCOhost)	((MH “Sinus Thrombosis+” OR MH “Cerebral Vein Thrombosis” OR TI (“cerebral venous thrombosis” OR “cerebral venous sinus thrombosis” OR “dural sinus thrombosis” OR CVST OR CVT) OR AB (“cerebral venous thrombosis” OR “cerebral venous sinus thrombosis” OR “dural sinus thrombosis” OR CVST OR CVT)) AND (MH “Pregnancy+” OR MH “Postpartum Period” OR TI (pregnan* OR postpartum OR puerper* OR peripartum OR gestation*) OR AB (pregnan* OR postpartum OR puerper* OR peripartum OR gestation*)) AND (TI (cohort OR longitudinal OR “follow up” OR registry OR retrospective OR prospective) OR AB (cohort OR longitudinal OR “follow up” OR registry OR retrospective OR prospective)) AND (TI (mortality OR recurrence OR anticoagulat* OR heparin OR enoxaparin OR obstetric* OR miscarriage OR stillbirth OR “live birth” OR preterm) OR AB (mortality OR recurrence OR anticoagulat* OR heparin OR enoxaparin OR obstetric* OR miscarriage OR stillbirth OR “live birth” OR preterm)))

### Data extraction

Data extraction was performed using a piloted, standardized form in order to achieve uniform capture of cohort structure, obstetric timing, management exposures, outcome denominators, and analytic population type. Study selection and data extraction were conducted independently by two reviewers. Disagreements were first resolved by discussion and consensus; when consensus could not be reached, a third reviewer was available for adjudication. Extracted data were cross-checked against tables, figures, and supplementary appendices in order to minimize transcription error. The following items were extracted: study identifiers (year, country/setting, design, recruitment/registry frame); cohort descriptors (acute pregnancy−/puerperium-associated CVST versus previous-CVST subsequent-pregnancy cohort; pregnancy versus postpartum strata; gestational week/postpartum day when reported); diagnostic confirmation criteria (MRI/MRV/CTV/DSA and defining imaging signs); anatomical distribution (sinus/vein segments; superficial versus deep system); clinical phenotype at presentation (headache, seizures, focal deficits, altered sensorium; NIHSS/GCS/mRS where available); etiologic and prothrombotic profiles (APS/aPL, inherited thrombophilia panels, anemia, infection, dehydration, hypertensive disorders, cesarean delivery, OCP/estrogen exposure, hyperhomocysteinemia and other biomarkers); baseline complications (ICH, venous infarction, edema, mass effect/herniation); management variables (initial anticoagulation type-UFH/LMWH/warfarin; dosing intensity; timing; monitoring; thrombolysis/endovascular when applicable; postpartum duration); and core outcomes (maternal mortality; functional outcomes; recurrent CVST; extra-cerebral VTE; live birth; miscarriage; stillbirth; preterm birth; placenta-mediated complications). The assessment window was explicitly abstracted as either the cohort assessment period or participant follow-up duration, while outcomes were stratified accordingly in order not to mix structurally different time bases.

### Risk-of-bias assessment

Risk of bias was assessed using the Newcastle–Ottawa Scale (12) for cohort studies, independently by two reviewers with disagreements resolved by consensus. Within the Selection domain, we appraised cohort representativeness, ascertainment of exposure including pregnancy/puerperium status and confirmation of CVST, demonstration that outcomes were not present at baseline where applicable, and cohort comparability. The Comparability domain considered adjustment or matching for key obstetric thrombosis-relevant confounders including thrombophilia, hypertensive disorders, infection, anemia, history of prior VTE, and anticoagulation exposure. The Outcome domain considered outcome ascertainment methods including medical records/registries, adequacy and duration of follow-up including ≥5 years, and completeness of follow-up.

### GRADE certainty assessment

The certainty of evidence for each core outcome was determined with GRADE (13), applied at the outcome level in combination with NOS judgments. Because the evidence base consisted of observational cohort studies, certainty began as low and was further downgraded for serious concerns regarding risk of bias (NOS limitations), inconsistency (unexplained heterogeneity across cohorts), indirectness (population/time-base mismatch between acute obstetric cohorts and subsequent-pregnancy cohorts), imprecision (wide confidence intervals and sparse events for recurrence/mortality), and suspected publication bias when asymmetry could be meaningfully evaluated. Upgrading criteria-large magnitude of effect, dose–response, or residual confounding likely minimizing observed effects-were considered if supported by coherent patterns across cohorts. Summary of Findings judgments were anchored to prespecified core outcomes (maternal mortality, recurrence, anticoagulation uptake, and key obstetric outcomes).

### Statistical analysis

Meta-analyses were planned and performed using the Meta-Analysis Online Software (14). For single-arm event outcomes (e.g., live birth, miscarriage, recurrent CVST, extra-cerebral VTE, maternal mortality, anticoagulation uptake), summary estimates were calculated as pooled event proportions using random-effects models to address heterogeneity between studies. Extra-cerebral VTE was defined as venous thromboembolism occurring outside the cerebral venous sinuses or cerebral veins. Variance-stabilizing transformation (logit scale) and suitable continuity corrections for sparse events were applied; study-level 95% CIs were calculated with binomial methods, and summary estimates were back-transformed to proportions. Heterogeneity was quantified using Cochran’s Q, I^2^ and τ^2^. Synthesis was stratified by assessment basis (previous-CVST subsequent-pregnancy follow-up cohorts versus acute pregnancy−/puerperium-associated CVST study-period cohorts) and by prespecified duration strata (e.g., 5–<10, 10–<14, ≥14 years) where applicable. No overall composite estimate was calculated across these two clinical populations because their denominators, outcome timing, and interpretive questions were not interchangeable. Formal publication-bias testing, including funnel-plot assessment, was not performed because fewer than ten studies contributed to each pooled endpoint and because sparse single-arm proportions make asymmetry tests unreliable. When comparative effect measures were available, pooling was planned on the log scale using generic inverse-variance methods under a random-effects model.

## Results

**Figure 1:**
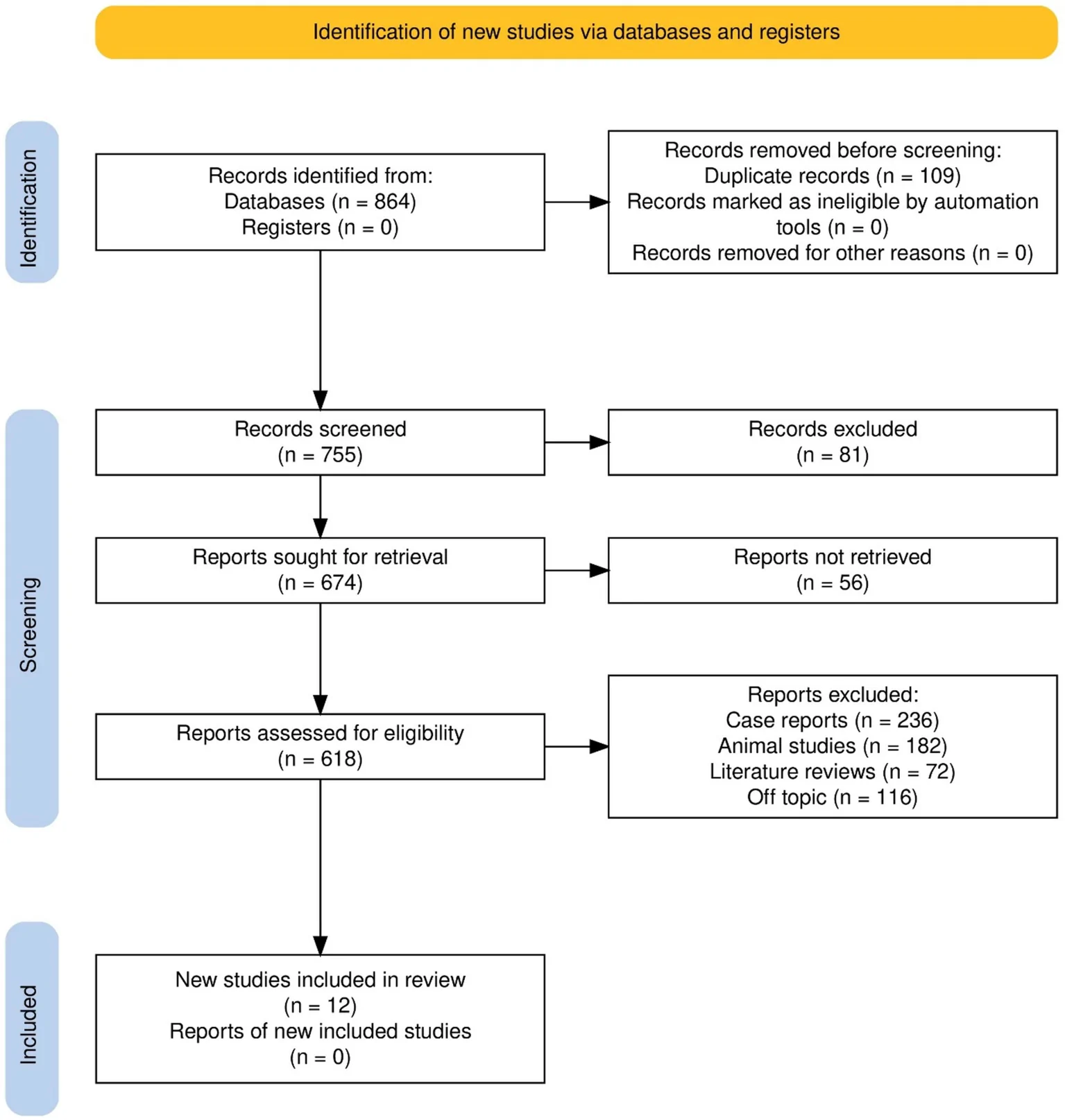
Study selection process for the review.

The literature search produced a total of 864 records; 109 duplicates were removed, and 755 titles/abstracts were screened (Figure 1). Thereafter, 81 records were excluded during screening due to full text unavailability, 674 reports were sought for retrieval, and 56 could not be retrieved due to paywall restrictions, leaving 618 full texts assessed for eligibility. Exclusions mainly consisted of case reports 236, animal studies 182, literature reviews 72, and off-topic records 116, thus leaving the review dataset with 12 eligible cohort studies (15–26).

### Bias levels assessed

**Figure 2:**
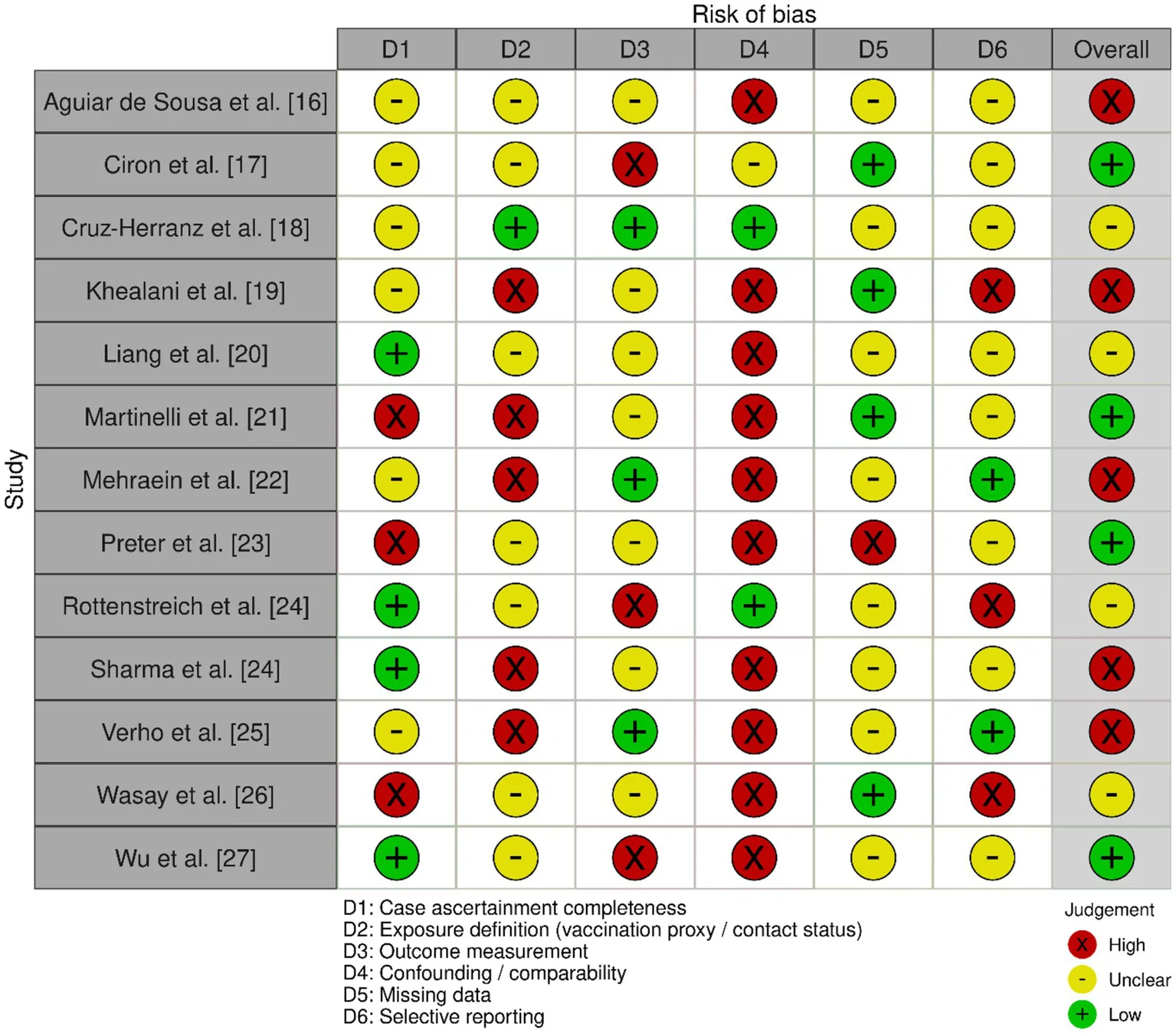
Bias levels assessed across the included studies.

Across the included cohorts (Figure 2), internal validity was primarily limited by confounding/comparability, with high risk present in the majority of datasets [notably in (15, 18–26)], reflecting incomplete adjustment for baseline risk structure, obstetric comorbidity, thrombophilia, and treatment selection. The completeness of case ascertainment ranged from high (20, 22, 25) to moderate (15–18, 21, 24) and low (19, 23, 26), reflecting heterogeneity in the comprehensiveness with which eligible CVST phenotypes were captured and verified. The exposure definition was frequently unclear or indirect in a number of cohorts (19, 22, 23, 25, 26), while outcome measurement was generally moderate-to-high but inconsistent [high in (16, 23, 26) versus low in (17, 21, 24)], implying differential granularity and reliability in how obstetric endpoints, recurrence, and functional outcomes were operationalized. Missing-data risk was generally unclear (15, 17, 19, 21, 23, 24, 26) yet was explicitly high in one dataset (22) and low in several (16, 18, 20, 25). Concerns about selective reporting were recurring [high in (18, 23, 25)] or vague in multiple cohorts (15–17, 19, 20, 22, 23, 26). Overall, risk-of-bias judgments clustered as high in five cohorts (15, 18, 21, 23, 24), moderate in four (17, 19, 23, 25), and low in four (16, 20, 22, 26), to indicate that pooled estimates derive from a mixed-certainty evidence base with confounding and reporting limitations as the principal threats to inference.

### Demographic variables assessed

The analysed studies (Table 2) ranged from 1996 to 2023 [1996 (22)–2023] and included geographic distributions across Europe [France (16, 22), Spain (17), Germany (21), Italy (20), Finland (24)] and Asia [China (19, 26), India (23), Pakistan/UAE (18), multicenter Asian network (25)], with additional multicenter registry-type cohorts [ISCVT (15); Israel (23)]. The eligible studies separated into two analytic populations: follow-up cohorts of women with previous CVT/CVST used to determine subsequent pregnancy outcomes or recurrence (15, 16, 21–23, 26), and acute pregnancy−/puerperium-associated CVST cohorts used to characterize acute presentation, management, and mortality (18, 19, 23, 25). One study was a nationwide register-based analysis (24), and one was a prospective, matched prophylaxis cohort followed through pregnancy and puerperium (20).

**Table 2:** Study-level design and follow-up characteristics of included cohorts.

Author ID	Year	Location	Study design	Sample size	Mean age (years)	Follow-up period
Aguiar de Sousa et al. (15)	2017	Multicenter (ISCVT women cohort)	Cohort follow-up of women with prior CVT; subsequent pregnancy outcomes	Women with prior CVT: n = 119; Women with ≥1 pregnancy: n = 47; Pregnancies: *n* = 82	Age at index CVT: 23 ± 5; Age at pregnancy: 30 ± 5	Median follow-up: 169 months (IQR 105–238)
Ciron et al. (16)	2013	France (regional, 4 hospitals)	Retrospective multicenter cohort + telephone follow-up	Women ≤40y with CVT: *n* = 62; Follow-up group: *n* = 60; Pregnancies: n = 45 (44 analyzed)	27.2 ± 6.7 at CVT	Mean 89.5 ± 60.6 months; median 76 months
Cruz-Herranz et al. (17)	2015	Spain	Observational cohort of young women with prior stroke/TIA; pregnancy outcomes (CVT subgroup included)	Total cohort: n = 102; CVT subgroup: n = 12; Pregnancies overall: *n* = 32 (in *n* = 27)	35.0 ± 7.5	Mean follow-up: 7.4 ± 5.6 years
Khealani et al. (18)	2008	Pakistan + UAE	Descriptive multicenter study; retrospective + prospective data	109	35.76 ± 14.05	Discharge + long-term FU: median 6 mo (range 3 d–5 y), available in 72 pts.
Liang et al. (19)	2017	China	Pregnancy-related CVT cohort	43	27.09 ± 5.09	Discharge outcomes + post-treatment sequelae described; duration NR
Martinelli et al. (20)	2016	Italy	Prospective cohort; first pregnancy after CVT (matched controls) with protocolized prophylaxis	CVT pregnancies: *n* = 52; Controls: *n* = 204	NR	Followed through pregnancy + puerperium (LMWH continued ≥6 weeks postpartum)
Mehraein et al. (21)	2003	Germany	Small cohort; recurrence risk during subsequent pregnancy/puerperium	Pregnancies: *n* = 22; Mean follow-up: 123 months	NR	Mean follow-up: 123 months
Preter et al. (22)	1996	France	Observational cohort (radiologically confirmed CVT; long-term follow-up)	77	39.0 ± 14.0	Mean 77.8 months
Rottenstreich et al. (23)	2019	Multicenter (Israel)	Retrospective cohort; pregnancy outcomes after prior CSVT	Women: *n* = 65; Pregnancies: *n* = 74	NR	Median CSVT→delivery interval: 1574 days; CSVT→first conception: 4.2 y (IQR 2.1–7.2)
Sharma et al. (23)	2019	Northeast India	Hospital-based CVT series	87	NR	In-hospital outcomes; deaths reported (9/87)
Verho et al. (24)	2023	Finland	Nationwide register-based cohort (pregnancy-associated stroke recurrence)	235	30.8 ± 5.4	Subsequent delivery follow-up: median 11.8 y (IQR 6.7–17.2)
Wasay et al. (25)	2012	12 centers, 8 Asian countries	Multicenter cohort of young Asian women (chart review; AIS vs. CVT predictors)	204 CVT (of 958 total)	NR (age modeled as <36 vs. ≥ 36)	mRS at admission/discharge/latest FU; 30-day FU available in 54/192 discharged
Wu et al. (26)	2020	Single-center (China)	Retrospective cohort; reproductive follow-up after CVST	Women: *n* = 72; Follow-up: *n* = 68; Subsequent pregnancies: *n* = 17 (in *n* = 13)	29.4 ± 5.9	Mean follow-up: 63.1 ± 46.8 months

CVST, cerebral venous sinus thrombosis; CVT, cerebral venous thrombosis; ISCVT, International Study on Cerebral Vein and Dural Sinus Thrombosis; FU, follow-up; IQR, interquartile range; NR, not reported; TIA, transient ischemic attack; LMWH, low-molecular-weight heparin.

The analytic units also differed between studies, including women-level cohorts [*n* = 62 (16)–119 (15)] and pregnancy-level cohorts [*n* = 17 pregnancies (26)–82 pregnancies (15)] in previous-CVST follow-up studies, whereas acute outcomes were primarily case-based [*n* = 43 (19)–235 (24)]. This denominator variation means that live birth, miscarriage, and recurrence estimates should be read as subsequent-pregnancy outcomes among selected women with prior CVST, while mortality and acute anticoagulation estimates should be read as acute-phase outcomes among women presenting with obstetric CVST, not as interchangeable components of a single pooled population (15–19, 21–26).

Long assessment windows were commonly reported as approximately 5–14 + years in previous-CVST follow-up cohorts [63.1 months (26), 77.8 months (22), 89.5 months (16), 123 months (21), 169 months (15), 11.8 years (24), 7.4 years (17)]. However, some acute pregnancy−/puerperium-associated CVST investigations employed short clinical ascertainment windows or did not report participant-level long-term follow-up [median 6 months (18), 30-day subset (25), duration not reported (19), in-hospital only (23)]. Thus, the ≥5-year criterion should be understood as a cohort assessment/study-period filter for acute outcomes and as a participant-level follow-up criterion mainly for recurrence and subsequent-pregnancy endpoints (15–26).

### Population timing and assessment framework

The clinical spectrum was mainly pregnancy–puerperium/postpartum, with puerperium constituting the larger component when quantified, 60.47% (19), and early postpartum clustering within 2 weeks (18) (Table 3). However, the population framework differed across datasets: some studies enrolled women with acute-onset pregnancy−/puerperium-associated CVST (18, 19, 23, 25), whereas others assessed pregnancies occurring after a previous CVT/CVST event (15, 16, 20, 21, 23, 26). Case definition was mainly imaging-confirmed using MRI/MRV [MRI 71% (18); MRV 66% (18)] with adjunct CT [CT 42% (18)] and rarely DSA [DSA 5% (18)]; explicit confirmation pathways using MRI/MRV with or without CTV/DSA were also mentioned (16, 25, 26).

**Table 3:** Clinical–diagnostic profile, risk architecture, imaging topography, and obstetric context across included CVST cohorts.

Author ID	Population type (pregnancy vs postpartum; gest/postpartum timing)	Diagnostic confirmation criteria (MRV/CTV/DSA; imaging signs)	CVST anatomical distribution (sinus/vein; superficial vs deep)	Clinical phenotype at presentation (key symptoms; scales)	Etiologic–prothrombotic profile + key labs/biomarkers	Baseline severity and complications (ICH/VI/edema/herniation)	Obstetric context/exposures
Aguiar de Sousa et al. (15)	Subsequent pregnancies after prior CVT: 82 pregnancies (47 women)	NR (ISCVT cohort; objective confirmation assumed)	NR	NR	Index CVT subtype: transient RF 70.6%, permanent RF 29.4% (pregnancy cohort)	NR	Pregnancy outcomes noted; obstetric mortality: 1 maternal death (eclampsia)
Ciron et al. (16)	Subsequent pregnancies after prior CVT: 45 total, 44 analyzed	Diagnosis confirmed by MRI ± venous MRA, contrast CT, arteriography, or combinations	Superficial sinus system predominated; deep system rarer (no segment % reported)	Headache 96.8%; ICHypertension 67.7%; seizures 32.3%; focal signs 25.8%; vigilance disorders 9.7%	Etiologies (%): OCP 79.0; hereditary thrombophilia 27.4; pregnancy 6.5; puerperium 4.8; neighboring infections 6.5; acquired thrombophilia 1.6 (others listed)	Acute outcomes: deaths 1.6%; discharge mRS median 0	Delivery among births: vaginal 22/24 (91.7%), CS 2/24 (8.3%); no preterm births
Cruz-Herranz et al. (17)	Pregnancy after cerebrovascular event (stroke/TIA); 32 pregnancies in 27 women (CVT subgroup *n* = 12)	NR	NR	NR	Antithrombotic use during pregnancy (overall): 17/32 (53.1%)	NR	Obstetric details NR
Khealani et al. (18)	Postpartum state 17% (abstract); detailed: 18/58 females postpartum (31%), 12/18 within 2 wks	Imaging used: MRI 71%, MRV 66%, CT 42%, DSA 5%, CTA	SSS 71%; transverse 47%; sigmoid 31%; straight 10%; cortical 6%; deep veins 3%	Headache 81%; focal deficits 45%; seizures 39%; mental status changes 37%	Predisposing: infection 18%; postpartum 17%; hyperhomocysteinemia 9%; genetic thrombophilia 5%; OCP 3%	66% infarction; 34% hemorrhagic infarct among infarcts	Hypercoag tests (performed/positives): homocysteine 35 tested, 29% positive; protein S 36, 8% pos; protein C 32, 6% pos; AT-III 27, 7% pos; LA 23, 4% pos
Liang et al. (19)	Pregnancy 17 (39.53%); puerperium 26 (60.47%)	Pregnancy-related CVT cohort; imaging distribution reported	SSS 65.12%; TS 51.16%; sigmoid 27.91%; straight 25.58%; deep veins 11.63%; cortical veins 13.95%; vein of Galen 4.65%	Headache 88.37%; seizures 39.53%; focal deficit 37.21%; altered sensorium 16.28%	Infection 30.23%; hypertensive disorders of pregnancy 16.28%; hyperhomocysteinemia 16.28%; hypercoagulability 11.63%	ICH 30.16%; cerebral infarction 25.58%; (HDP/seizure/ICH associated with higher mRS/death)	Lipids often abnormal: TG abnormal 18.60% (mean 4.78 mmol/L in abnormal group); CHO abnormal 65.12% (mean 6.72 mmol/L)
Martinelli et al. (20)	First pregnancy after CVT: 52 pregnancies (CVT group)	Objective CVT diagnosis at index event	Prior CVT: TS 84.6%; SSS 50.0%; sigmoid 46.1%; straight 9.6%; cortical 11.5%; deep 5.8%; ≥2 sinuses 38.4%	NR	RF: OCP 80.7%; pregnancy/puerperium 17.3%; inherited thrombophilia 53.8% (FVL 19.2%; prothrombin 23.1%; protein C 5.8%; protein S 11.5%; AT 1.9%); APLAS 1.9%	NR	Obstetric outcomes tracked; late obstetric complications 24.0% (CVT group)
Mehraein et al. (21)	Subsequent pregnancies after prior CVST: 22 pregnancies; recurrence 0	Objective confirmation (angiography/MR-angiography per study)	NR	NR	Outcomes summary: miscarriages 3/22 (1 voluntary, 2 spontaneous); uneventful 16/22; live births 19/22	Recurrence 0/22	Obstetric context NR
Preter et al. (22)	Obstetric cause suspected: 9/77 (11.7%)	CT/MRI lesions listed; venous occlusion sites tabulated	SSS/LS 72 (93.5%); straight 11 (14.3%); cortical veins 24 (31.2%); deep veins 7 (9.1%)	Focal signs 47 (61.0%); ICHT 30 (39.0%); seizures 28 (36.4%); altered consciousness 22 (28.6%)	Suspected causes: systemic 44.1%; local 10.4%; obstetric 11.7%; infectious 5.2%; OCP 7.8%; unknown 20.1%	Parenchymal lesions: none 46.8%; isolated hypo 16.9%; isolated hyper 13.0%; both 9.1%	NR
Rottenstreich et al. (23)	74 pregnancies after prior CSVT	NR	Prior CSVT distribution: lateral sinus 84.6%; sigmoid 67.7%; SSS 27.7%; straight 7.7%; deep system 6.2%	NR	Thrombophilia 43.1% (prothrombin mutation 15.4%; FVL 6.2%; protein S def 6.2%; APLAS 7.7%); estrogen-related RF 80%	ICH at index CSVT 6.2%	Gestational age at delivery median 38 wks; delivery mode: vaginal 44/54, CS 10/54 (CS group higher); preterm 5/54
Sharma et al. (23)	Predominantly puerperium (discussion: CVT ~ 13 × more common puerperium vs. pregnancy)	Confirmed after MRI/MRV; DSA if needed	SSS 57.4%; R-TS 17.24%; L-TS 10.34%; R-sigmoid 8.04%; L-sigmoid 5.74%; internal cerebral vein 1.14%	Headache 73.5%; vomiting 60.6%; focal seizures 8%; generalized seizures 2.29%; altered sensorium 6.89%	Cesarean delivery predictor 50.5%; hypertension “strong predictor” (GTN/PE/Eclampsia listed)	Parenchymal involvement (venous infarct + ICH) 55.17%	Anemia common: hypochromic 61%, normochromic 29%; leukocytosis 43.4%
Verho et al. (24)	Pregnancy-associated stroke cohort; prior CVT subgroup *n* = 13	Stroke subtype includes CVT (as definition)	NR	NR	Prior CVT group described; prophylactic LMWH used in 11 pregnancies	Recurrent PAS after subsequent delivery: 4/73 (5.5%) including recurrent CVT (*n* = 1)	NR
Wasay et al. (25)	Pregnancy 9 (5%); postpartum 40 (19%) among CVT (*n* = 204)	“Confirmed by CT or MRI/MRV”	Location data (subset n = 125): SSS alone 35%; TS alone 11%; SS alone 5%; cortical 1%; superficial+deep 8%	Severity scores reported (mRS at admission/discharge) (NIHSS/GCS NR)	Hypercoagulopathy 47 (23%); OCP 20 (10%); infection 7 (3%); cancer 3 (2%); unidentified 70 (35%)	CT/MRI: hemorrhagic infarcts 22%; non-hemorrhagic infarcts 21%; edema 14%; mass effect 10%; midline shift 3%	Thrombophilia panel abnormal rates among tested: protein C 46%, protein S 48%, AT-III 40%, homocysteine 43%, aPL 18%
Wu et al. (26)	Subsequent pregnancies after CVST: 17 pregnancies (13 women)	CVST confirmed by MRI/MRV and/or DSA/CTV (study diagnostic work-up)	TS 76.4%; sigmoid 69.4%; SSS 55.6%; straight sinus 11.1%; deep venous thrombosis 11.1%; cortical vein 8.3%	Headache 91.7%; focal deficits 41.7%; seizures 30.6%; visual impairment 26.4%; coma 4.2%	RF: anemia 31.9%; infection 15.3%; pregnancy/puerperium 12.5%; OCP 6.9%; preeclampsia 2.8%; thrombophilia 4.2% (APS 1.4%)	ICH 23.6%; SAH 8.3%; venous infarction 15.3%; edema 26.4%; herniation 4.2%	In pregnancy outcomes: PIH 11.8%; GDM 5.9%; delivery: CS 6/10, vaginal 4/10; preterm 2/10

APS/APLAS, antiphospholipid syndrome; aPL, antiphospholipid antibodies; AT/AT-III, antithrombin (III); CTV, CT venography; DSA, digital subtraction angiography; FVL, Factor V Leiden; GCS, Glasgow Coma Scale; GDM, gestational diabetes mellitus; GTN, gestational hypertension; HDP, hypertensive disorders of pregnancy; ICH, intracranial hemorrhage; ICU, intensive care unit; LA, lupus anticoagulant; MRV/MRA, MR venography/MR angiography; mRS, modified Rankin Scale; NIHSS, National Institutes of Health Stroke Scale; OCP, oral contraceptive pill; PAS, pregnancy-associated stroke; PE, preeclampsia; PIH, pregnancy-induced hypertension; SAH, subarachnoid hemorrhage; SSS, superior sagittal sinus; TS, transverse sinus; SS, sigmoid sinus; VI, venous infarction.

### Anatomy and phenotype

A superficial sinus predominance was observed, with frequent SSS involvement [SSS 71% (18); 65.12% (19); 57.4% (23)] and substantial involvement of the transverse/sigmoid region [TS 51.16% (19); sigmoid 31% (18)], whereas deep venous involvement remained comparatively low [deep veins 3% (18); 11.63% (19)]. Clinically, headache predominated [headache 81% (18); 88.37% (19); 91.7% (26); 96.8% (16)], with notable occurrences of seizures [seizures 39% (18); 39.53% (19)] and focal deficits [focal deficit 45% (18); 37.21% (19)].

### Prothrombotic architecture and obstetric context

The architecture of risk combined estrogen-related exposure [OCP 79.0% (16); estrogen-related risk factors 80% (23)] with thrombophilia [inherited thrombophilia 53.8% (20); thrombophilia 43.1% (23)], alongside infection [infection 18% (18); 30.23% (19)] and hypertensive disorders [16.28% (19)]. Complications indicated meaningful baseline injury, including intracerebral hemorrhage [ICH 30.16% (19); 23.6% (26)] and infarction [cerebral infarction 25.58% (19)] with edema [edema 26.4% (26)].

### Anticoagulation strategy and intensity

Across pregnancies following a prior CVST event, the uptake of antithrombotic prophylaxis was generally high [83.1% (15); 90.9% during pregnancy (23)], with predominant use of low-molecular-weight heparin (LMWH)/enoxaparin [LMWH 67.6% among prophylaxed pregnancies (15); enoxaparin 91.9% of pregnancies (23)] and predominantly prophylactic dosing [prophylactic 79.4% of enoxaparin-exposed pregnancies (23)] (Table 4). Protocolized initiation occurred early in gestation [median 7 weeks (20)] with continuation through at least 6 weeks postpartum (20). In contrast, other cohorts reported heterogeneous patterns of low-dose heparin or lack of prophylaxis in a substantial fraction (21), and very limited anticoagulation exposure during pregnancy in some series [2 of 17 pregnancies (26)]; within acute obstetric CVST cohorts, therapeutic anticoagulation was commonly administered [67% (18)] and heparin treatment was frequent [80.5% (22)], while post-discharge regimens included warfarin [48% (25)] and antiplatelet therapy (25). Extractable anticoagulation-related adverse-event data, including major bleeding and intracranial bleeding attributable to therapy, were not consistently available across the included cohorts; therefore, a pooled safety analysis for bleeding outcomes was not performed.

**Table 4:** Management pathways and maternal outcomes.

Author ID	Initial anticoagulation strategy (UFH/LMWH; dose/monitoring; timing)	Care setting/process metrics (ICU; time-to-dx/AC)	Maternal mortality endpoints + functional outcomes	Recurrence & longer-term outcomes (CVST/VTE; persistent deficits)
Aguiar de Sousa et al. (15)	Thromboprophylaxis in 68/82 (83.1%); LMWH in 46/68 (67.6%)	NR	Maternal deaths: 1 (eclampsia, 38 wks)	Recurrent CVT: 1/82 (1.2%) at 10 wks; extra-cerebral VTE: 2/82 (2.4%)
Ciron et al. (16)	Most frequent prophylaxis: LMWH in last trimester + puerperium (6–8 wks postpartum); early losses occurred without prophylaxis	Diagnostic delay at index CVT: 8.3 ± 7.6 days; median 6 days	Maternal mortality during subsequent pregnancies: 0; long-term mRS median 0	Pregnancy recurrence: 1 CVT recurrence in 44 pregnancies; extra-CVT during pregnancy: 0
Cruz-Herranz et al. (17)	Antithrombotic therapy during pregnancy (overall): 17/32 (53.1%)	NR	Recurrent stroke during pregnancy: 0/32 (overall); maternal mortality NR	Recurrence during pregnancy: none reported (overall cohort)
Khealani et al. (18)	Therapeutic anticoagulation 96/109 (67%); acute anticoagulation 73/109 (67%)	Diagnostic delay: onset → diagnosis median 10 d; admission → diagnosis median 1 d	In-hospital deaths 7/109	Poor outcome at discharge: 43 + deaths 7 (≈46% dead/dependent)
Liang et al. (19)	Anticoagulation in most; 2 puerperium cases with mild symptoms received symptomatic care only (no anticoagulant)	NR	Deaths n = 5 (in cohort of 43)	Higher mRS associated with infection, seizure, ICH, HDP
Martinelli et al. (20)	Protocol: LMWH prophylaxis in all CVT pregnancies (started median 7 wks); continued through puerperium ≥6 wks	NR	Maternal deaths: 0; thrombotic events: 0	Recurrent thrombosis (CVT/VTE): 0/52
Mehraein et al. (21)	Low-dose heparin prophylaxis in 5 pregnancies (timing varied: full pregnancy+puerperium in 2; puerperium only in 2; 2nd half+puerperium in 1); none in remaining 14 continuing pregnancies	NR	Maternal deaths: 0; maternal thrombosis recurrence: 0	Recurrence: 0/22 pregnancies
Preter et al. (22)	Heparin treatment 62/77 (80.5%)	NR	NR	Long-term sequelae tabulated (e.g., seizures, neuropsychiatric)
Rottenstreich et al. (23)	Anticoagulation during pregnancy: 50/55 (90.9%); enoxaparin 68/74 pregnancies (91.9%)—prophylactic 54/68 (79.4%), intermediate 8/68 (11.7%), therapeutic 6/68 (8.8%)	NR	Maternal mortality: 0	Recurrent CSVT during pregnancy: 0; follow-up VTE events: 11/65 (16.9%)
Sharma et al. (23)	Anticoagulation during pregnancy (number unspecified)	NR	In-hospital mortality: 9/87 (10.34%)	NR (mRS distribution NR)
Verho et al. (24)	Prophylactic LMWH used in 11 pregnancies among prior-CVT women	Subsequent delivery follow-up median 11.8 y	Recurrent PAS after subsequent delivery 4/73 (5.5%) (includes recurrent CVT *n* = 1)	NR
Wasay et al. (25)	Discharge antithrombotic: warfarin 94/192 (48%); aspirin 46 (24%); other antiplatelets 28 (15%)	30-day FU: available 54/192; 1 additional death before 30-day FU	Mortality CVT 4.5% (overall); obstetric vs. non-obstetric mortality 4% vs. 5%	Prognosis: mRS 0–2 in 74% (CVT); discharge mRS distribution reported (subset n = 130)
Wu et al. (26)	During pregnancy: anticoagulation used in 2/17 pregnancies (both “no live birth” group)	NR	Maternal mortality during pregnancy: NR; functional outcomes reported overall (not pregnancy-specific)	Recurrent CVST during pregnancy: 1/17 (5.9%) in APS; after therapeutic LMWH → induced abortion

CVT/CVST cerebral venous (sinus) thrombosis; SSS superior sagittal sinus; TS transverse sinus; MRV magnetic resonance venography; CTV CT venography; DSA digital subtraction angiography; VI venous infarction; ICH intracerebral hemorrhage; APS antiphospholipid syndrome; OCP oral contraceptive pills; PIH pregnancy-induced hypertension; GDM gestational diabetes; LMWH low-molecular-weight heparin; mRS modified Rankin Scale; NR not reported.

### Care-process metrics

Diagnostic delay was not trivial when measured, with a median index-event delay of approximately 6 days (16) and a median onset-to-diagnosis interval of about 10 days (18), while the admission-to-diagnosis latency was short [median 1 day (18)], indicating pre-hospital recognition intervals probably dominated the total time-to-diagnosis in acute presentations (18).

### Maternal death and functional outcomes

Maternal mortality in subsequent pregnancies after prior CVST was rare to absent [0 deaths (16); 0 (20); 0 (21); 0 (23)] with one reported death attributed to eclampsia at 38 weeks (15). In contrast, acute obstetric CVST cohorts demonstrated non-negligible in-hospital mortality [7/109 (18); 9/87 (23)] and an overall mortality signal approaching 4.5% in a broader CVST cohort (25). The included studies did not consistently report the exact interval between CVST diagnosis and maternal death; mortality was generally reported as in-hospital, discharge, or 30-day mortality, which precluded reliable identification of the highest-risk hour-, day-, or week-level interval after diagnosis. Functional recovery tended to be favorable in long-term pregnancy-after-CVST cohorts [modified Rankin Scale [mRS] median 0 (16)]; however, acute series demonstrated a substantial proportion of patients dead or dependent at discharge [approximately 46% (18)], while another dataset reported mRS 0–2 in 74% (25).

### Recurrence and longer-term outcomes

Recurrent CVST in subsequent pregnancy or puerperium was uniformly low, although not universally zero. Across cohorts with extractable pregnancy-level data, three recurrent CVST events were identified across 291 subsequent pregnancies [1/82 (15), 1/44 (16), 0/52 (20), 0/22 (21), 0/74 (23), and 1/17 (26)]. Extra-cerebral VTE during pregnancy/puerperium was less consistently reported; the ISCVT pregnancy cohort reported 2 extra-cerebral VTE events among 82 pregnancies (15), whereas several other cohorts reported no events or did not separate extra-cerebral VTE as an independent pregnancy-period outcome. Outside pregnancy during longer follow-up, Rottenstreich et al. reported 11 VTE events among 65 women with previous CSVT (23), and Verho et al. reported recurrent pregnancy-associated stroke in 4/73 subsequent deliveries, including one recurrent CVST event (24). Long-term anticoagulation exposure outside pregnancy was inconsistently reported, limiting pooled interpretation of recurrence in relation to prophylaxis or extended anticoagulation. Follow-up time for recurrent CVST and extra-cerebral VTE was not uniformly reported at the outcome level in the individual studies; when unavailable, it was explicitly treated as not reported and the recurrence estimates were interpreted as study-reported events within each available cohort assessment period rather than as time-standardized incidence rates.

### Meta-analysis observations

Pooled outcomes are presented below by analytic population and should not be interpreted as a single merged estimate for all obstetric CVST. Live birth, miscarriage, and recurrence estimates came from follow-up cohorts of women with previous CVST who subsequently became pregnant, whereas maternal mortality and acute anticoagulation uptake came from acute pregnancy−/puerperium-associated CVST cohorts. The observation periods and denominators therefore differed by endpoint, and the clinical interpretation is population-specific.

**Figure 3:**
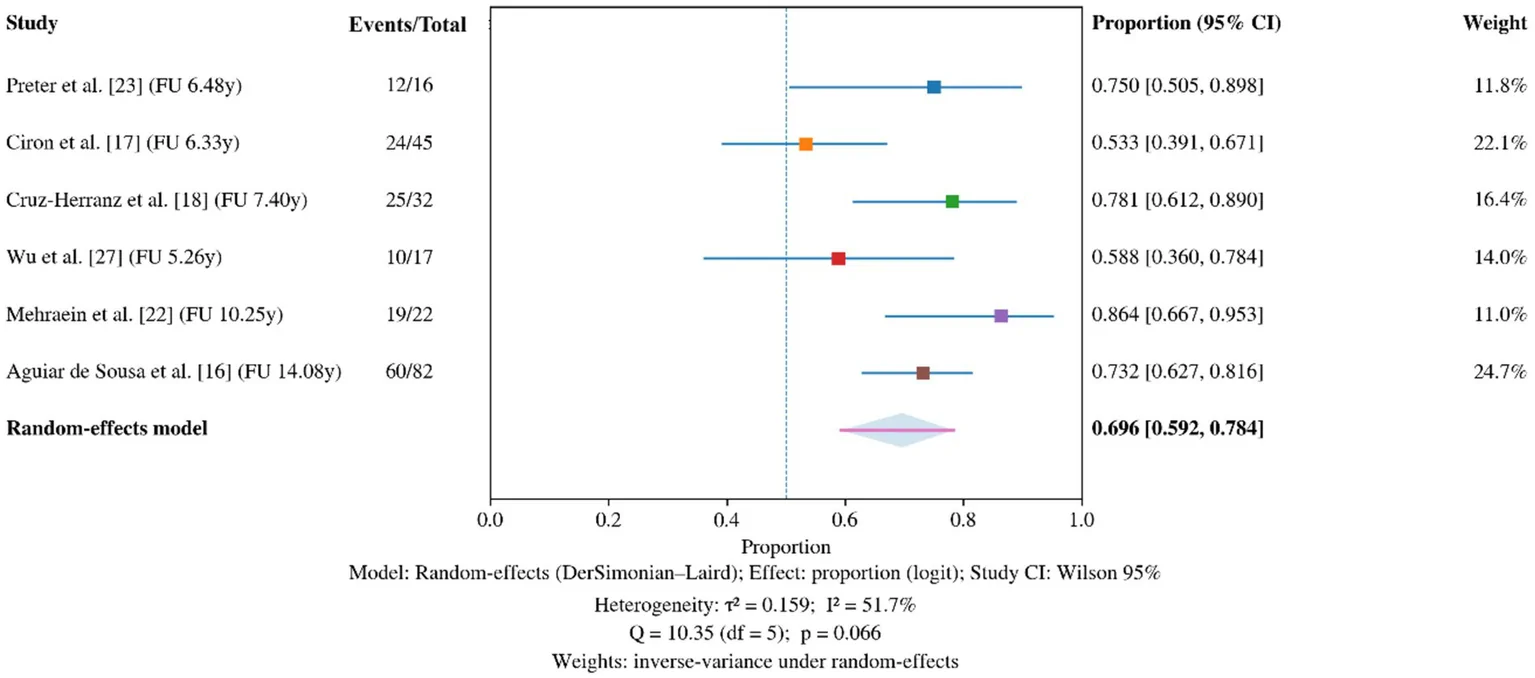
Forest plot of live births/deliveries after subsequent pregnancy in women with prior CVST—follow-up cohorts ≥5 years. CI, confidence interval; CVST, cerebral venous sinus thrombosis; df, degrees of freedom; FU, follow-up; *I*^2^, Higgins’ heterogeneity statistic; *p*, *p*-value; *Q*, Cochran’s *Q* statistic; RE, random-effects; Weight (%), inverse-variance random-effects weight; y, years; *τ*^2^, between-study variance.

Figure 3 shows that, within previous-CVST follow-up cohorts evaluating subsequent pregnancy, the pooled proportion of live births/deliveries was 69.6% (95% CI 59.2–78.4%). Heterogeneity between studies was moderate (I^2^ = 51.7%, τ^2^ = 0.159; Q = 10.35, df = 5, *p* = 0.066), indicating meaningful variation in delivery rates across cohorts, with greater dispersion in smaller studies and tighter estimates in larger cohorts. This estimate applies to subsequent pregnancies after prior CVST and should not be generalized to acute pregnancy−/puerperium-associated CVST presentations.

**Figure 4:**
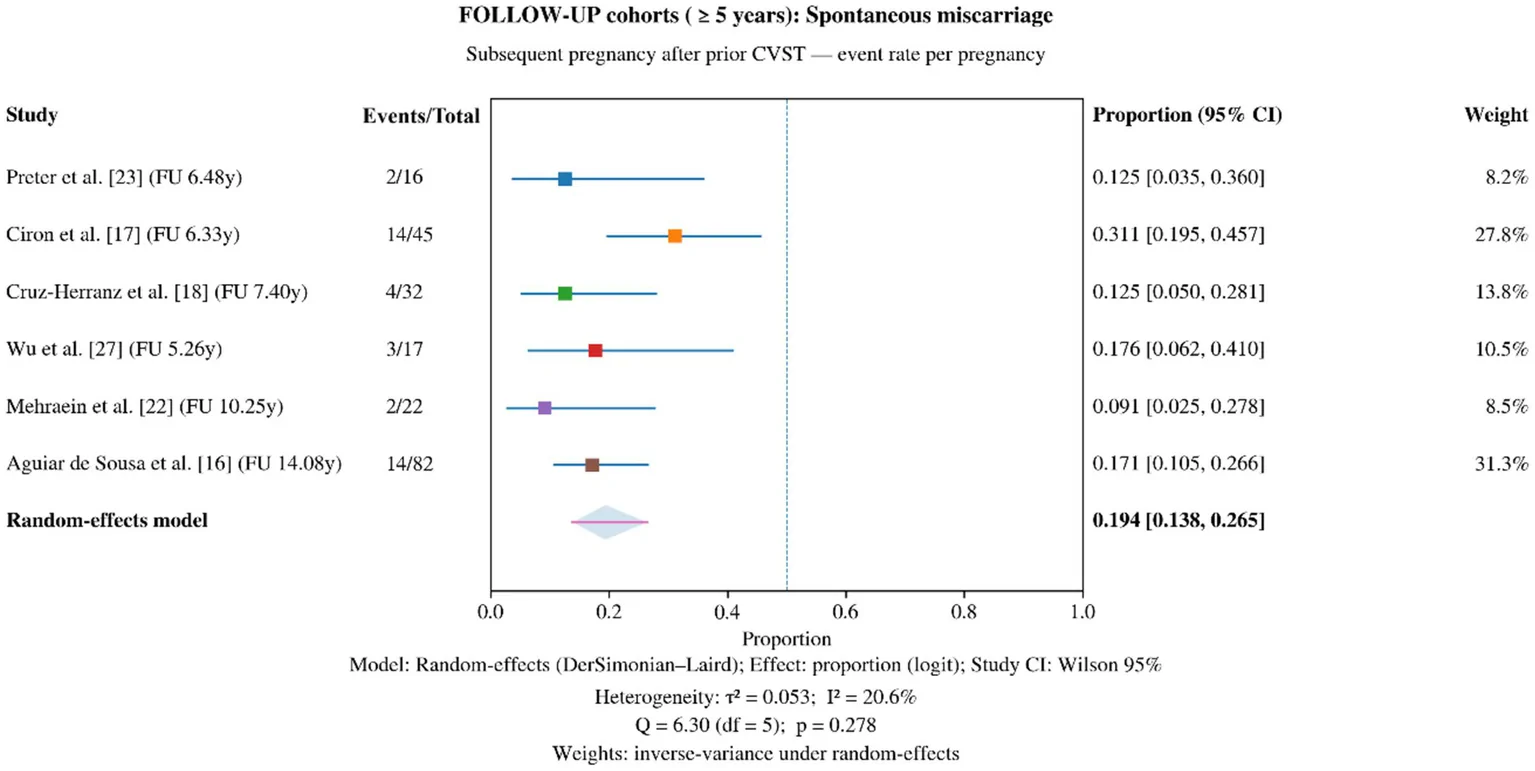
Forest plot of spontaneous miscarriage after subsequent pregnancy in women with prior CVST—follow-up cohorts ≥5 years. CI, confidence interval; CVST, cerebral venous sinus thrombosis; *df*, degrees of freedom; FU, follow-up; *I2*, Higgins’ heterogeneity statistic; *p*, *p*-value; *Q*, Cochran’s *Q* statistic; RE, random-effects; Weight (%), inverse-variance random-effects weight; y, years; *τ2*, between-study variance.

The pooled proportion of spontaneous miscarriage in previous-CVST subsequent-pregnancy follow-up cohorts was 19.4% (95% CI 13.8–26.5%), as represented in Figure 4. Heterogeneity was low (I^2^ = 20.6%, τ^2^ = 0.053; Q = 6.30, df = 5, *p* = 0.278), suggesting that miscarriage rates were relatively consistent across these follow-up cohorts and that most variability was compatible with sampling error.

**Figure 5:**
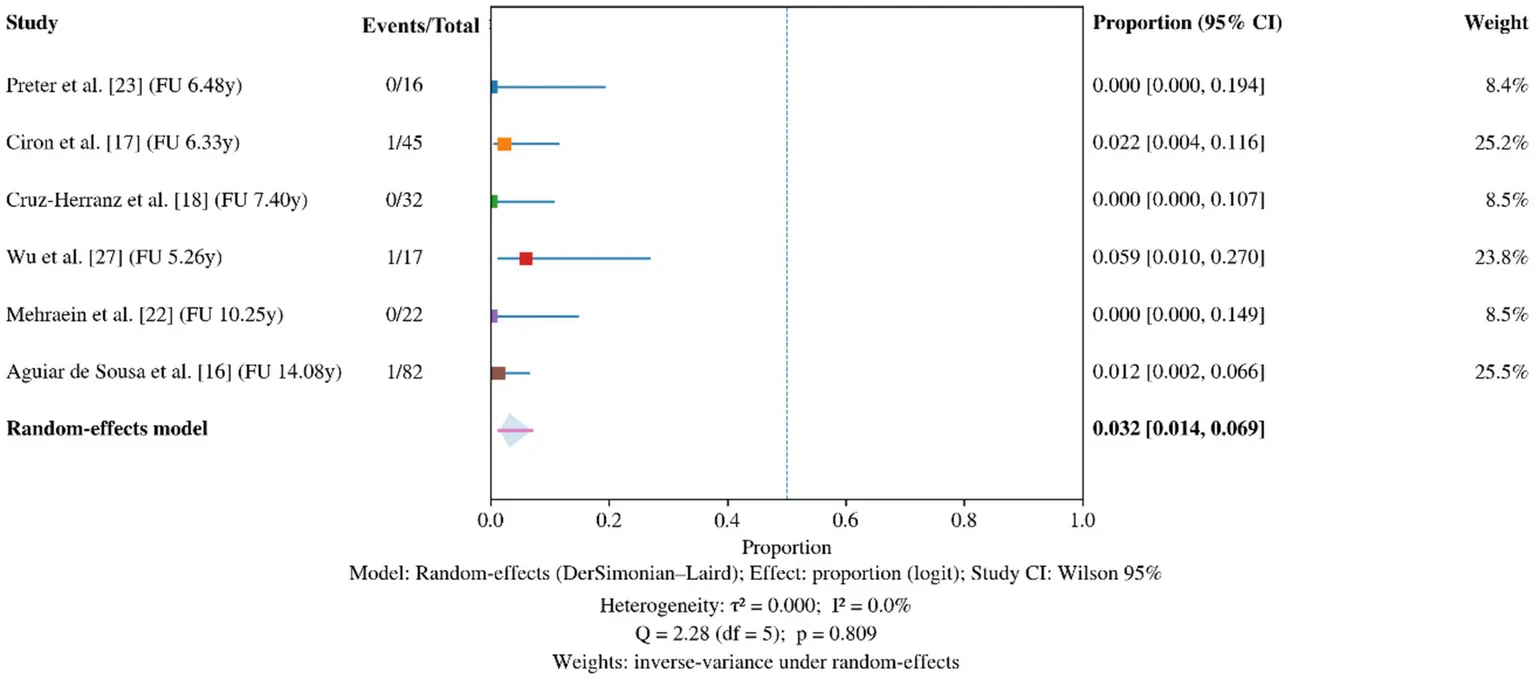
Forest plot of recurrent CVST during subsequent pregnancy/puerperium in women with prior CVST—follow-up cohorts ≥5 years. CI, confidence interval; CVST, cerebral venous sinus thrombosis; df, degrees of freedom; FU, follow-up; I^2^, Higgins’ heterogeneity statistic; *p*, *p*-value; *Q*, Cochran’s *Q* statistic; RE, random-effects; Weight (%), inverse-variance random-effects weight; *y*, years; *τ*^2^, between-study variance.

Figure 5 displays that recurrent CVST in a subsequent pregnancy or puerperium after previous CVST was infrequent, with a pooled estimate of 3.17% (95% CI 1.43–6.89%). Heterogeneity was absent (I^2^ = 0%, τ^2^ = 0.000; Q = 2.28, df = 5, *p* = 0.809), implying a uniformly low recurrence signal across selected survivor cohorts; however, interpretation remains limited by sparse event counts, prophylaxis variation, and possible selection of women considered suitable for pregnancy.

**Figure 6:**
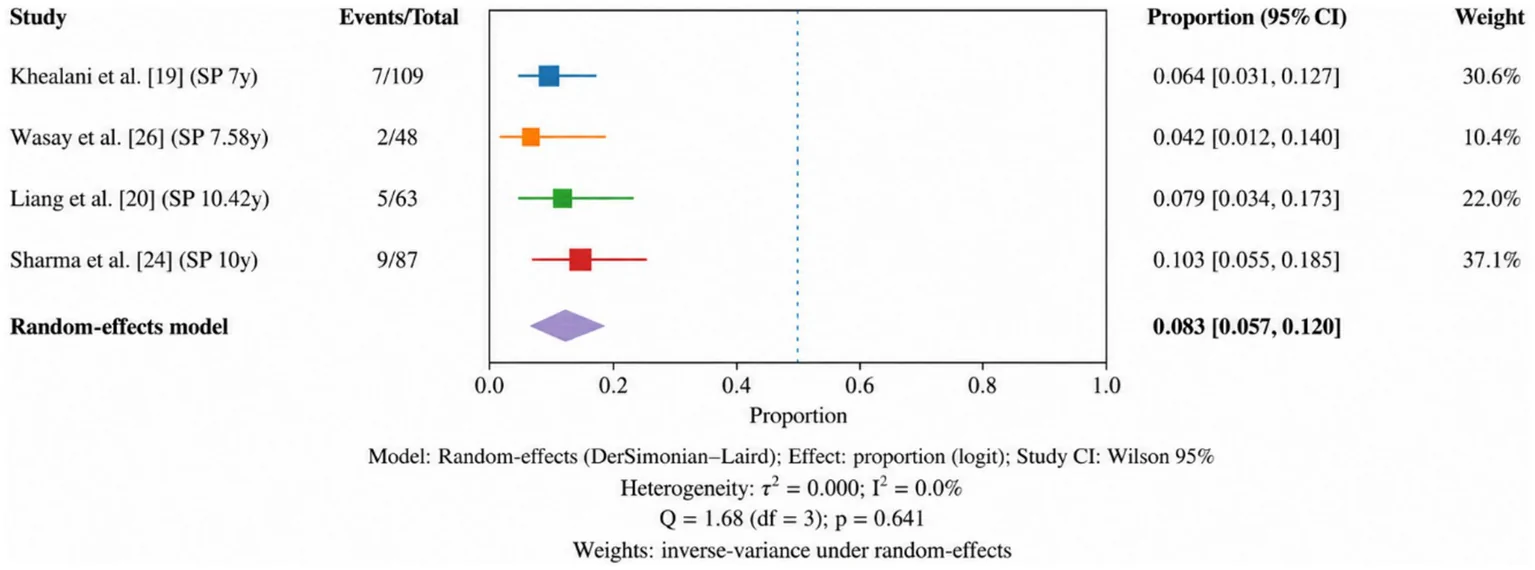
Forest plot of maternal mortality in acute pregnancy/puerperium CVST cohorts—study period ≥5 years. CI, confidence interval; CVST, cerebral venous sinus thrombosis; df, degrees of freedom; SP, study period; *I*2, Higgins’ heterogeneity statistic; *p*, *p*-value; *Q*, Cochran’s *Q* statistic; RE, random-effects; Weight (%), inverse-variance random-effects weight; y, years; *τ*2, between-study variance.

Maternal mortality among pooled acute obstetric CVST cases (Figure 6) was 8.28% (95% CI 5.65–11.97%). No statistical heterogeneity was observed (I^2^ = 0%, τ^2^ = 0.000; Q = 1.68, df = 3, *p* = 0.641), suggesting that the case-fatality estimates of included acute cohorts were directionally consistent across settings and study periods. This estimate reflects acute case fatality among women presenting with pregnancy−/puerperium-associated CVST and is not comparable to maternal mortality during subsequent pregnancy after prior CVST, where deaths were rare or absent.

**Figure 7:**
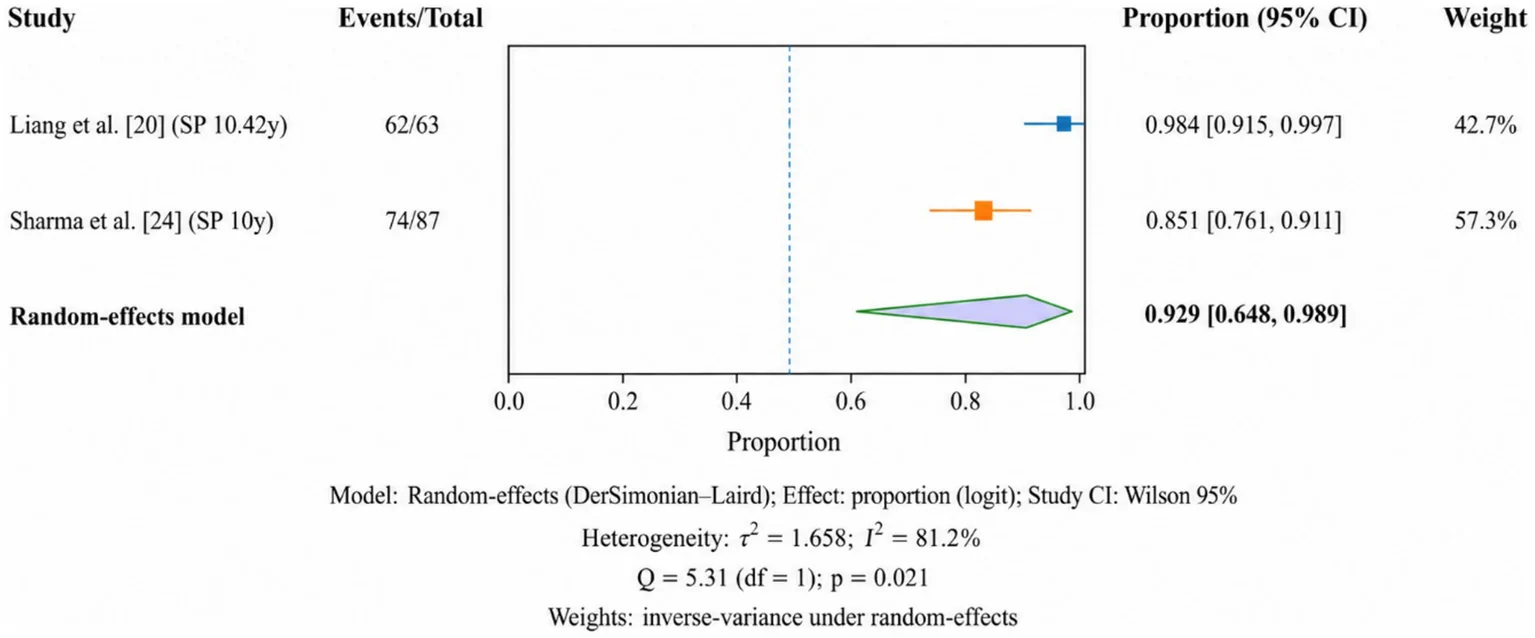
Forest plot of anticoagulation use in acute pregnancy/puerperium CVST cohorts- study period ≥5 years. CI, confidence interval; CVST, cerebral venous sinus thrombosis; df, degrees of freedom; SP, study period; I^2^, Higgins’ heterogeneity statistic; p, p-value; Q, Cochran’s Q statistic; RE, random-effects; Weight (%), inverse-variance random-effects weight; y, years; τ^2^, between-study variance.

Figure 7 shows that anticoagulation use in acute obstetric CVST cohorts was high overall, with a pooled estimate of 92.9% (95% CI 64.8–98.9%), yet the estimate was highly heterogeneous (I^2^ = 81.2%, τ^2^ = 1.658; Q = 5.31, df = 1, *p* = 0.021). Potential sources of this heterogeneity include geographic differences in acute care pathways, variable adoption of guideline-based anticoagulation, differential access to neurologic and obstetric critical-care resources, and incomplete reporting of dose intensity, treatment timing, and postpartum continuation. This pooled estimate should be interpreted as acute treatment uptake, not as prophylaxis use during subsequent pregnancies after prior CVST.

The overall key statistics pertaining to the meta-analysis are reported in Table 5, with each outcome explicitly assigned to its source population and observation window.

**Table 5:** Summary of key pooled clinical outcomes by analytic population and observation window.

Outcome	Pooled estimate	95% CI	Heterogeneity	Clinical interpretation
Live births/deliveries after subsequent pregnancy (previous-CVST follow-up cohorts)	69.6%	59.2–78.4%	I^2^ = 51.7%	Most subsequent pregnancies ended in live birth/delivery, but estimates varied moderately across follow-up cohorts and should not be generalized to acute obstetric CVST presentations.
Spontaneous miscarriage after subsequent pregnancy (previous-CVST follow-up cohorts)	19.4%	13.8–26.5%	I^2^ = 20.6%	Miscarriage estimates were relatively consistent across previous-CVST follow-up cohorts.
Recurrent CVST during subsequent pregnancy/puerperium (previous-CVST follow-up cohorts)	3.17%	1.43–6.89%	I^2^ = 0%	Recurrence was uncommon in selected survivor cohorts, supporting individualized counseling rather than an assumption that future pregnancy is universally risk-free.
Maternal mortality in acute pregnancy/puerperium-associated CVST cohorts	8.28%	5.65–11.97%	I^2^ = 0%	This estimate reflects acute case fatality among women presenting with obstetric CVST, not outcomes of later pregnancies after prior CVST.
Anticoagulation use in acute pregnancy/puerperium-associated CVST cohorts	92.9%	64.8–98.9%	I^2^ = 81.2%	Use was high overall but highly heterogeneous across acute cohorts and care settings; it should not be interpreted as prophylaxis uptake in subsequent pregnancies after prior CVST.

### Certainty bias assessment

The certainty of evidence was low across core endpoints because the included studies were predominantly observational cohorts with residual concerns regarding selection processes, confounding control, and outcome ascertainment (Tables 6, 7 respectively). Live birth/delivery and miscarriage outcomes were rated as low certainty and applied specifically to previous-CVST subsequent-pregnancy cohorts: both were directly applicable to that counseling question and had acceptable precision, but residual bias and cohort design limitations precluded upgrading. Recurrent CVST during subsequent pregnancy or puerperium was rated as very low certainty because events were sparse and the estimate was sensitive to small changes in event counts. Maternal mortality in acute obstetric CVST cohorts was rated as low certainty because the mortality signal was consistent but remained limited by observational design, modest event counts, and the study-period rather than participant-level meaning of the ≥5-year criterion. Anticoagulation uptake was rated as very low certainty because few acute cohorts provided extractable treatment counts and between-cohort heterogeneity was substantial.

**Table 6:** GRADE evidence profile.

Evidence domain (outcome set)	Cohorts (k)	Design class	Methodological limitations	Heterogeneity across cohorts	Applicability to the review question	Precision of estimate	Small-study/reporting bias	Overall confidence (GRADE)
Live births/deliveries after subsequent pregnancy (prior CVT/CVST cohorts) (15–17, 21, 22, 26)	6	Observational cohort	Serious (retrospective elements, incomplete adjustment; outcome ascertainment variability)	Serious (moderate heterogeneity)	Not serious (direct population/outcome; long follow-up aligned)	Not serious (reasonable N; CIs acceptable)	Undetected/unclear	Low
Spontaneous miscarriage after subsequent pregnancy (prior CVT/CVST cohorts) (15–17, 21, 22, 26)	6	Observational cohort	Serious	Not serious (low heterogeneity)	Not serious	Not serious	Undetected/unclear	Low
Recurrent CVT/CVST during pregnancy/puerperium (prior CVT/CVST cohorts) (15–17, 21, 22, 26)	6	Observational cohort	Serious	Not serious (minimal heterogeneity)	Not serious	Very serious (sparse events; wide relative uncertainty)	Undetected/unclear	Very low
Maternal mortality in acute pregnancy/puerperium CVST cohorts (18, 19, 23, 25)	4	Observational cohort	Serious (hospital-based selection; limited confounding control)	Not serious (minimal heterogeneity)	Not serious (direct acute obstetric CVST endpoint)	Serious (event counts modest; CI width relevant)	Undetected/unclear	Low
Anticoagulation uptake in acute pregnancy/puerperium CVST cohorts (extractable treatment counts) (19, 23)	2	Observational cohort	Serious	Very serious (substantial heterogeneity; practice variation)	Not serious	Very serious (k small; CI wide)	Likely	Very low

**Table 7:** Summary of findings.

Endpoint	Absolute effect expected (pooled proportion; per 100)	Comparative metric	Participants (cohorts)	Certainty (GRADE)
Live births/deliveries after subsequent pregnancy (prior CVT/CVST cohorts) (15–17, 21, 22, 26)	69.6 per 100 (95% CI 59.2 to 78.4)	Not applicable (single-arm proportion)	214 pregnancies (6 cohorts)	Low
Spontaneous miscarriage after subsequent pregnancy (prior CVT/CVST cohorts) (15–17, 21, 22, 26)	19.4 per 100 (95% CI 13.8 to 26.5)	Not applicable	214 pregnancies (6 cohorts)	Low
Recurrent CVT/CVST during pregnancy/puerperium (prior CVT/CVST cohorts) (15–17, 21, 22, 26)	3.2 per 100 (95% CI 1.4 to 6.9)	Not applicable	214 pregnancies (6 cohorts)	Very low
Maternal mortality in acute pregnancy/puerperium CVST cohorts (18, 19, 23, 25)	8.3 per 100 (95% CI 5.7 to 12.0)	Not applicable	307 CVST cases (4 cohorts)	Low
Anticoagulation use in acute pregnancy/puerperium CVST cohorts (extractable treatment counts) (19, 23)	92.9 per 100 (95% CI 64.8 to 98.9)	Not applicable	150 CVST cases (2 cohorts)	Very low

## Discussion

The association between CVST in pregnancy and the puerperium reflects an important clinical intersection between venous thromboembolism physiology and neuroobstetrical medicine, in which patient outcome hinges on early detection, prompt imaging, anticoagulation, and organized reproductive follow-up (27). Frameworks delineating pregnancy-associated stroke suggest that CVST generally emerges as a consequence of overlapping risk states, especially hypertensive disorders, whereas review articles centered on obstetric CVST underscore the role of pregnancy-induced hypercoagulability in combination with factors like dehydration, infection, anemia, operative vaginal delivery, and hormonal exposures (28, 29). In addition, neurology-based systematic reviews point out the urgency of symptoms such as headache, seizures, visual disturbances, and focal neurological abnormalities, since diagnosis delay could delay anticoagulation, even though effective therapy is available (30, 31).

This review revealed two clinically different patterns that should remain analytically separate. First, among selected women with prior CVST who subsequently conceived, live birth/delivery was common and recurrent CVST during pregnancy or the puerperium was uncommon. Second, among women presenting with acute pregnancy−/puerperium-associated CVST, acute maternal mortality remained clinically meaningful despite frequent anticoagulation use. These findings should not be collapsed into a single message of either reassurance or risk: prior-CVST subsequent-pregnancy cohorts inform counseling and prophylaxis planning, whereas acute obstetric CVST cohorts inform emergency recognition, acute treatment quality, and maternal survival.

Current CVST research reveals that older data should be viewed with caution due to differential precipitating factor profiles, obstetric approaches, thrombophilia screening, infection trends, and accessibility to advanced imaging and endovascular salvage (32, 33). As mentioned before, a reproductive-context approach is critical since pregnancy, puerperium, and oral contraceptive use represent biologically coherent high-risk windows linked to hormonal and prothrombotic effects (34). Nevertheless, reproductive context must be interpreted together with cohort design: a postpartum acute presentation, a remote index CVST followed by later pregnancy, and a registry-defined subsequent delivery are not equivalent data structures. Moreover, reports from resource-poor countries have demonstrated that late presentation, lack of MRV, anemia, and infection can modify complication patterns and functional prognosis, making it challenging to generalize findings based on pooled results (10).

As mentioned before, anticoagulation stands out as a major management domain with marked heterogeneity. In acute obstetric CVST cohorts, anticoagulation uptake was high but variably reported, and differences in geography, local guideline implementation, neuroimaging access, critical-care availability, hemorrhagic lesion management, dose intensity, and treatment duration may all influence outcomes. In previous-CVST subsequent-pregnancy cohorts, the relevant question is different and concerns prophylactic strategy, initiation timing, postpartum continuation, and recurrence prevention. For these reasons, standardized protocols should separately address acute therapeutic anticoagulation for active CVST and prophylactic anticoagulation for women with previous CVST who are planning or undergoing pregnancy.

Based on our risk-of-bias and GRADE analyses, one may state that pooled results are likely to be clinically valuable but hardly definitive due to the observational design, limited number of studies, absence of randomization and control groups, and limited adjustment for important variables. In particular, most outcomes were collected in observational cohorts, and confounding was addressed inadequately, with insufficient accounting of thrombophilia, hypertension, infections, anemia, and treatment bias. Hence, the relatively low recurrence rate cannot be regarded as conclusive evidence that future pregnancies are entirely safe under any circumstances; it is likely that personalized management and thromboprophylaxis is necessary in many cases.

In terms of broad venous thrombosis literature, even for neonatal cerebral sinovenous thrombosis, current evidence highlights that these conditions are imaging-dependent and underdiagnosed, thus demanding diagnostic criteria standardization (35). The narrative regional cohort experiences confirm that incidence and management depend not only on physiology but also on imaging capacity, surveillance frequency, hospitalization rates, and local referral practices (36–39). Therefore, clinical decision-making regarding CVST during pregnancy must incorporate a multidisciplinary approach considering maternal stabilization, fetal health, delivery planning, and postpartum care (40). Neuro-ophthalmology and emergency neurology guidelines recommend imaging studies when papilledema, visual symptoms, severe headache, seizures, and/or focal neurological deficits are present during late pregnancy or the puerperium (41, 42).

Systems-of-care strategies involving stroke-in-pregnancy pathways, in which consultation triggers and anticoagulation protocols have been established, are fundamental for avoiding diagnostic delays and inconsistent practices (43). In regard to rare infectious etiologies, such as meningoencephalitis with associated venous sinus thrombosis, one must take into account secondary causes in atypical presentations (44). Current stroke-in-pregnancy evidence indicates the importance of systematic postpartum follow-up to ensure adequate anticoagulation, avoid complications, and manage risk factors (45, 46).

The approach to interpreting rare neurovascular disease in pregnancy may also gain from understanding adjacent conditions whose evidence base is similarly limited. Recently published studies on brain arteriovenous malformations in pregnancy underscore the need for rare neurovascular diseases to involve data pooling, literature review, multidisciplinary decision-making, personalized mother and baby risk assessment, and interpretive caution due to the timing, referral patterns, and management cut-off points being different among centers (47). Though the pathophysiology of bAVM and CVST differ, there are some methodological difficulties that pertain to the current review such as small sample sizes, heterogeneity in observation periods, framing of risk factors related to the pregnancy state, and difficulty distinguishing between the acute phase of maternal risk and later reproductive counseling.

Clinically, women with a past history of CVST and pregnant status should undergo a thorough pre-conception counseling, evaluation of continuing thrombophilia or severity of the event and develop an adequate prophylaxis strategy. The prevention of CVST via low-molecular weight heparin in pregnancy period and its continuation as a prophylaxis after delivery, especially for at least 6 weeks if required, agrees with scientific evidence and common practices in the considered cohorts. On the contrary, obstetric CVST requires urgent medical attention: sudden headache, seizure, papilledema, neurological deficit, changes in consciousness or other symptoms should raise the alertness about venous imaging and timely anticoagulation therapy unless contraindicated.

### Limitations

Observational cohort data comprised the evidence base, precluding causal inference and leaving open the possibility of residual confounding related to the thrombophilia, hypertensive disorder, infection, anemia, and choice of treatment. Differing outcome definitions and denominators were used across the cohorts, and several outcome measures were defined by few studies with rare events, thus creating imprecision and sensitivity to changes in the number of events. An important consideration in this review is that two different groups of people were analyzed: cohorts of patients with CVST during acute phase of pregnancy/puerperium and the previous-CVST group with analysis continuing into pregnancy. While analyses were stratified, understanding must be made that live births, miscarriages, and recurrences are outcomes of subsequent pregnancies, while maternal mortality and anticoagulation use are acute phase outcomes. There were differences in diagnostic and management reporting across the cohorts, and heterogeneous anticoagulation strategies implied a case-specific care and lack of generalizability. The requirement for ≥5-year follow-up made recurrence and subsequent pregnancy outcomes more interpretable, while there was a weaker reason for acute maternal mortality or acute anticoagulation use. It probably decreased the number of available studies, excluded the useful short series and preferred centers with better follow-up or registry system. Moreover, 56 full-text articles could not be accessed because of paywalls, thus selection bias might be present. In addition, women with severe CVST during the index event might be less likely to try for subsequent pregnancies, resulting in their underrepresentation in the subsequent pregnancy cohorts.

### Clinical recommendations

Routine reporting of sinus topography, deep-system involvement, hemorrhagic lesions, treatment timing, anticoagulation dose intensity, postpartum duration, and functional outcomes should be standardized to improve comparability. Future studies should explicitly label whether the cohort represents acute pregnancy−/puerperium-associated CVST or subsequent pregnancy after previous CVST and should report denominators as women, pregnancies, deliveries, or acute CVST cases as appropriate. Risk stratification frameworks should integrate modifiable triggers, including infection control, blood-pressure optimization, correction of anemia, prevention of dehydration, and avoidance of estrogen-related exposures where feasible, alongside thrombophilia evaluation when clinically justified. For women with prior CVST contemplating pregnancy, counseling should convey that delivery outcomes were commonly favorable and recurrence was infrequent in selected cohorts, while emphasizing individualized prophylaxis planning and postpartum continuation when indicated. In acute obstetric CVST, anticoagulation protocols should be harmonized across centers to reduce variability, and future cohorts should report bleeding complications, recurrent CVST, extra-cerebral VTE, mortality timing, and functional outcomes using standardized scales.

## Conclusion

This synthesis should be interpreted through two distinct clinical lenses. In previous-CVST follow-up cohorts, future pregnancy was generally associated with favorable obstetric outcomes and low recurrence when women received structured counseling, individualized risk stratification, and appropriate prophylactic management. In acute pregnancy- or puerperium-associated CVST cohorts, the evidence addressed emergency diagnosis, acute anticoagulation, functional outcome, and maternal mortality, with a measurable mortality burden persisting across included studies. Therefore, the review supports separate reporting of acute obstetric CVST and subsequent pregnancy after prior CVST, standardized venous imaging pathways, consistent heparin-based acute treatment protocols, individualized prophylaxis for future pregnancies, careful postpartum continuation, and harmonized outcome reporting.

## Data Availability

The original contributions presented in the study are included in the article/supplementary material, further inquiries can be directed to the corresponding author.
